# Predicting student self-efficacy in Muslim societies using machine learning algorithms

**DOI:** 10.3389/fdata.2024.1449572

**Published:** 2024-12-13

**Authors:** Mohammed Ba-Aoum, Mohammed Alrezq, Jyotishka Datta, Konstantinos P. Triantis

**Affiliations:** ^1^Department of Industrial and Systems Engineering, Virginia Tech, Blacksburg, VA, United States; ^2^Department of Industrial and Systems Engineering, King Fahd University of Petroleum and Minerals, Dhahran, Saudi Arabia; ^3^Department of Statistics, Virginia Tech, Blacksburg, VA, United States

**Keywords:** academic performance, educational equity, machine learning, Muslim societies, self-efficacy, self-regulation, socio-emotional learning, student wellbeing

## Abstract

**Introduction:**

Self-efficacy is a critical determinant of students' academic success and overall life outcomes. Despite its recognized importance, research on predictors of self-efficacy using machine learning models remains limited, particularly within Muslim societies. This study addresses this gap by leveraging advanced machine learning techniques to analyze key factors influencing students' self-efficacy.

**Methods:**

An empirical dataset collected was used to examine self-efficacy among secondary school students in Muslim societies. Four machine learning algorithms-Decision Tree, Random Forest, XGBoost, and Neural Network-were employed to predict self-efficacy using two demographic variables and 10 socio-emotional, cognitive, and regulatory factors. The predictors included culturally relevant variables such as religious/spiritual beliefs and collectivist-individualist orientation. Model performance was assessed using root mean square error (RMSE) and r-squared (*R*^2^) metrics to ensure reliability and validity.

**Results:**

The results showed that Random Forest outperformed the other models in accuracy, as measured by *R*^2^ and RMSE metrics. Among the predictors, self-regulation, problem-solving, and a sense of belonging emerged as the most significant factors, contributing to more than half of the model's predictive power. Other variables such as gratitude, forgiveness, empathy, and meaning-making displayed moderate predictive value, while gender, emotion regulation, and collectivist-individualist orientation had minimal impact. Notably, religious/spiritual beliefs and regional factors showed negligible influence on self-efficacy predictions.

**Discussion:**

This study enhances the understanding of factors influencing self-efficacy among students in Muslim societies and offers a data-driven foundation for developing targeted educational interventions. The findings highlight the utility of machine learning in education research, demonstrating its ability to uncover insights for equitable and effective decision-making. By emphasizing the importance of regulatory and socio-emotional factors, this research provides actionable insights to elevate student performance and well-being in diverse cultural contexts.

## 1 Introduction

Self-efficacy is defined as a person's belief in their ability to complete specific tasks or achieve specific goals (Bandura, 1997). It is a central construct in social cognitive theory, positively and strongly correlated with an individual's cognitive and behavioral engagement in a given task. Self-efficacy affects how people think, feel, and act. It also has an impact on a person's decision to take action, the types of goals and activities they pursue, and the amount of effort, persistence, and time they are willing to devote to completing a task (Bandura, 2006, 2018). Numerous studies support the assertion that a person's belief in their ability to complete a task has a greater impact on success than actual capability (Bandura, 1986). While people cannot complete tasks that are beyond their abilities simply by believing they can, self-efficacy beliefs can function as internal rules that individuals follow to determine the amount of effort, persistence, and perseverance required to achieve a goal optimally. Researchers have investigated the impact of self-efficacy on these variables and found significant relationships (Pajares, 1996).

In educational settings, self-efficacy is one of the most fundamental mechanisms influencing a student's learning experience, academic achievement, and future success (Edgar et al., 2019; Usher and Pajares, 2008; Zimmerman, 2000). Prior studies have provided strong evidence that self-efficacy is a positive predictor of motivation to learn and perform across different academic areas and levels. Self-efficacy can influence the instigation, direction, persistence, and outcomes of achievement-related actions (Schunk and Pajares, 2002). It has likewise shown a significant positive association with student engagement and attention (Caraway et al., 2003). It also affects students' decision-making and level of resilience in the face of challenges, which affects the degree of success they are likely to experience in the future (Bandura, 1986). At the college level, studies have shown that undergraduate students with high self-efficacy beliefs stay in their majors longer (Lent et al., 1984) and consider a broader range of future career options than those with lower self-efficacy perceptions (Church et al., 1992). Poor self-efficacy beliefs often undermine students' motivation and can lead to task avoidance, passivity, lack of task engagement, and resignation, which can make failure unavoidable (Bandura, 1997; Pajares, 1996). Students with low self-efficacy are also more likely to experience mental and behavioral problems and drop out of school, jeopardizing their future academic and career opportunities (Schwarzer and Luszczynska, 2006).

Self-efficacy is a crucial factor in psychological and educational research due to its established links with educational and health outcomes, such as academic achievement, performance, and wellbeing (Coutinho, 2008; Pajares, 2008). However, assessing self-efficacy in Muslim societies presents unique technical challenges that stem from cultural, social, and systemic factors specific to these contexts. Although self-efficacy is widely studied, there is a notable gap in rigorous machine learning research focused on self-efficacy as a primary predictive variable (Tan and Cutumisu, 2022). Cultural values significantly shape self-efficacy beliefs, with collectivist orientations, high power distance, and social restraint often associated with lower self-efficacy levels, emphasizing the need for cultural sensitivity when assessing this construct (Jin et al., 2023). In Muslim societies, self-efficacy is embedded in collective values that prioritize spirituality, community, and respect for hierarchical relationships, contrasting with the individual autonomy and achievement focus common in Western contexts like the United States, Canada, and France (Kagitcibasi, 2005; Nasser et al., 2019). Additionally, limited empirical research on self-efficacy has been conducted in Muslim-majority regions, where educational challenges are substantial and development is often constrained by a lack of socio-emotional studies that could improve education at both individual and systemic levels (Nasser et al., 2019). While countries such as Afghanistan, Iraq, and Pakistan have received international support for advancing primary and secondary education, these initiatives have shown limited impact on expected outcomes due to underlying cultural and social obstacles (Bureau for Policy and Program Coordination, 2004; Colclough et al., 2010). For example, while net school enrollment has increased, many students still lack basic skills even after years of schooling, indicating a discrepancy between schooling and learning (Filmer et al., 2018). Furthermore, local opposition to externally funded education initiatives often arises when these efforts disregard indigenous religious practices and cultural traditions, which underscores the need for culturally adapted educational interventions (Hargreaves et al., 2001; Kapoor, 2014; Sahlberg et al., 2017). Addressing these contextual challenges in self-efficacy research within Muslim societies could improve research validity and encourage local adoption of development programs (Abu-Nimer and Nasser, 2017).

The purpose of this study was to predict student self-efficacy in Muslim societies, utilizing machine learning algorithms that incorporated a holistic set of 10 factors. These factors included empathy, forgiveness, sense of belonging, problem-solving, meaning-making, gratitude, self-regulation, emotion regulation, religion/spirituality, and a collectivist cultural orientation. Additionally, two demographic attributes, namely region and gender, were incorporated into the model. By analyzing these multifaceted dimensions, this study sought to enhance our understanding of the factors that influence student self-efficacy in Muslim societies. The study was guided by the following research questions:

1) What are the factors that are most important in predicting the self-efficacy of secondary school students in Muslim societies?
2) Which machine learning model has the best predictive performance for self-efficacy in this particular context?

The dataset and survey used to answer these questions were collected and provided by the International Institute of Islamic Thought (IIIT) as part of an initiative to advance education and human development in Muslim communities. This survey, which focuses on K-12 and university students and teachers in Muslim-majority societies, offers a comprehensive understanding of diverse values and competencies, including socio-emotional and cognitive traits that are often overlooked in mainstream research.

By making data-driven predictions on students' self-efficacy and considering constructs such as empathy, forgiveness, moral reasoning, and community-mindedness—which are not only universal but also deeply resonate with Islamic values—this research aimed to bridge a research gap, providing insights that could lay the foundation for more contextual and effective educational interventions in Muslims societies. This study adds to the body of scientific knowledge on self-efficacy, socio-emotional learning, and the application of machine learning. By delivering data-driven insights into factors that contribute to student self-efficacy, the study could enrich and advance theoretical models of self-efficacy. Moreover, it broadens the application of machine learning algorithms by predicting student self-efficacy in a novel context. The predictive model might also help in identifying students with relatively lower or higher levels of self-efficacy and devising strategies to enhance their self-efficacy and academic achievement.

## 2 Literature review

### 2.1 Theoretical background on self-efficacy

Bong and Clark (1999) suggested that self-efficacy is primarily a cognitive judgment of one's own abilities rather than an emotional response toward oneself. This evaluation considers multiple sources of information and assigns varying degrees of importance to them, leading to the formation of a perception of one's own capability. A foundational work on self-efficacy was structured by Bandura (1997), which theorized that self-efficacy beliefs develop based on students' interpretation of information from four sources: mastery experience, vicarious experience, verbal persuasion, and emotional state. The most influential of these is usually mastery experience or previous performance (Bandura, 1986, 1997). Successful experience raises mastery expectations and confidence, whereas failure lowers them, especially if the failures occur early in the learning journey. For instance, after students complete an assigned academic activity, they assess the results, and based on these interpretations, judgments of competence are created or changed. If students believe their efforts have been successful, their confidence to complete similar or related tasks increases. On the other hand, if they believe their efforts have not produced the effect they desired, their confidence in their ability to succeed in similar endeavors decreases. The negative impact of occasional failure is likely to be reduced after strong self-efficacy beliefs are developed through repeated success. Indeed, failures that are later overcome by determined effort can strengthen self-motivated persistence if it is discovered through experience that even the most difficult obstacles can be overcome by sustained effort. The effects of failure on self-efficacy depend in part on when the failures happen and how they fit into the overall pattern of experiences (Bandura, 1977).

Self-efficacy beliefs are most likely to change during skill development as an individual faces novel tasks (Bandura, 1997). When students notice a gradual improvement in their skills over time, their self-efficacy beliefs are typically boosted, even if failure occurs periodically. When students overcome obstacles or complete difficult tasks, experiences that lead to mastery are especially powerful in enhancing a person's self-efficacy (Bandura, 1997). Also, experienced mastery in a domain often has long-term effects on one's self-efficacy. Students who have excelled in a subject throughout their education are likely to believe they can continue to excel in that subject for many years to come (Usher and Pajares, 2008). Furthermore, once enhanced self-efficacy is established in one area, it tends to extend to other situations where performance had previously been debilitated by an obsession with personal shortcomings (Bandura, 1997). Thus, improvements in behavior can transfer not only to similar situations but also to activities that are significantly different from those the treatment was focused on (Bandura, 1977).

Vicarious experience from observing others is another source for building self-efficacy. Students usually evaluate their own performance and abilities in relation to the accomplishments of others (Bandura and Barab, 1973). For instance, they compare their exam results with those of classmates to interpret their own scores. If a student discovers that the majority of classmates received lower scores, their self-efficacy will most likely increase. On the other hand, if most of their classmates received higher scores, their confidence would most likely be reduced (Usher and Pajares, 2008). Another vicarious experience influencing self-efficacy is through social models, particularly when students are unsure of their own abilities or have little prior experience with the task at hand. For instance, when a student sees a classmate succeed in a challenging task, they might develop expectations that they too can do it if they intensify and persist in their efforts. Modeling, on the other hand, may undermine an observer's confidence, particularly if the model fails at a task perceived to be simple (Usher and Pajares, 2008). The degree to which students identify with the model in the relevant area determines how much of an impact the model's success or failure has on them (Schunk, 1987). Vicarious experience is normally less effective than mastery experience in influencing self-efficacy beliefs (Bandura, 1977).

Verbal and social persuasion is the third major influence on students' self-efficacy (Bandura, 1977). Encouragement from trusted parents, teachers, and peers can boost students' confidence in their academic abilities. Students who are persuaded verbally that they have the ability to master a given task are more likely to mobilize and sustain effort in the classroom than those who harbor self-doubt. Verbal persuasion does not have a significant effect on long-term persistence, but it can motivate immediate help for students to overcome self-doubt. Also, people who are socially convinced that they can handle difficult situations and are given provisional aids for effective action are more likely to exert more effort than those who only receive performance aids (Bandura, 1977).

The fourth source of self-efficacy, according to Bandura (1977) model, is the student's physiological and psychological state, which can be affected by anxiety, stress, fatigue, and mood. Emotional state is not only a crucial component of wellbeing but also of self-efficacy and the way individuals perceive themselves and have faith in their ability to achieve their goals. Exposure to high levels of stress and anxiety while performing an activity increases the likelihood that students will underperform on a task and can affect their confidence. In contrast, feelings of belonging, satisfaction, and happiness increase self-efficacy beliefs. In general, improving students' physical and emotional wellbeing and decreasing negative emotional states should boost self-efficacy. Self-regulation is another trait that has been linked to self-efficacy in various studies (Schunk and Zimmerman, 2008; Zimmerman, 1995). Self-regulation is a process and skill that enables individuals to proactively manage their circumstances and environment, as well as personally activate and control their cognitions, emotions, and behaviors, in order to successfully complete certain tasks and achieve their own goals, according to social cognitive theory (Bandura, 1977, 1986).

### 2.2 Empirical and machine learning studies on self-efficacy

Numerous studies have explored the relationship between self-efficacy and a range of personal and educational factors through regression analysis or structural equation modeling (see Table 1). Using regression analysis, DeWitz et al. (2009) identified a significant correlation between general self-efficacy and college students' sense of purpose in life. Meanwhile, McMahon and Wernsman (2009) used hierarchical linear regression to assess how classroom environment and a sense of school belonging affected academic self-efficacy. Their study indicated that both these factors significantly predicted academic self-efficacy, with the classroom environment being a more influential factor. Notably, Rey (2009) highlighted a gap in research focusing on positive psychological aspects, such as gratitude, and its link to self-efficacy. That study revealed a direct correlation; students who felt more gratitude also felt more self-efficacious. On the other hand, Saroughi and Kitsantas (2021) used structural equation modeling to examine the relationships between personal (e.g., self-efficacy for learning, self-regulation), contextual (e.g., stereotype threat, sense of belonging), and wellbeing (e.g., negative affect, positive affect, academic satisfaction, life satisfaction) variables. Their work showed that sense of belonging and stereotype threat directly predicted student self-efficacy and emphasized the mediating role of self-efficacy between feelings of belonging, stereotype threat, and academic satisfaction. Similarly, Rakhshanderou et al. (2021) used structural equation modeling to shed light on the connection between spirituality and self-efficacy, suggesting that fostering spiritual aspects can fortify students' confidence in their abilities. Collectively, these findings illuminate the multifaceted drivers of self-efficacy, ranging from inner sentiments of gratitude to broader educational contexts.

**Table 1:** Summary of empirical and machine learning studies on self-efficacy.

**References**	**Objective**	**Method**	**Major finding**
McQuiggan et al. (2008)	Model self-efficacy using physiological data.	Experimental design (33 students), Naive Bayes, Decision Tree	Built two sets of self-efficacy classification models (high vs. low), and accuracy ranged from 82.1% to 87.3%.
Rey (2009)	Examine the relationship between positive psychological constructs and self-efficacy.	Regression modeling	Gratitude was a significant predictor of self-efficacy.
McMahon and Wernsman (2009)	Investigate the relationship between classroom environment, school belonging, and academic self-efficacy.	Hierarchical linear regression	Classroom environment and school belonging significantly predicted academic self-efficacy, with classroom environment being the stronger predictor.
DeWitz et al. (2009)	Explore the relationship between self-efficacy beliefs and purpose in life.	Regression modeling	Self-efficacy was the most significant predictor of purpose in life scores.
Ezen-Can and Boyer (2014)	Classify students based on self-efficacy and collected natural language utterances.	K-medoids clustering algorithm	Utterance use differed between students with high and low self-efficacy.
Rizk and Farooque (2021)	Assess computer science students' self-efficacy and categorize it as low, medium, or high.	Factor analysis, k-nearest neighbors	Problem-solving was the most important predictor of self-efficacy, followed by college satisfaction.
Rakhshanderou et al. (2021)	Analyze the predictive role of spirituality in self-efficacy among college students	Structural equation modeling	Spirituality was found to be a positive predictor of self-efficacy among college students.
Saroughi and Kitsantas (2021)	Examine the relationships between personal factors (e.g., self-efficacy, self-regulation), contextual factors (e.g., sense of belonging), and wellbeing.	Structural equation modeling	Sense of belonging and stereotype threat directly predicted self-efficacy, which mediated relationships between sense of belonging, stereotype threat, and academic satisfaction.
Tan and Cutumisu (2022)	Model self-efficacy based on test performance and responses to a survey for international student assessment.	Random forest and XGBoost	Most important predictors were meaning in life and motivation to master tasks.

Recently, machine learning has increasingly been utilized to predict student academic outcomes and wellbeing through self-efficacy. However, only a few studies have prioritized predicting students' self-efficacy. McQuiggan et al. (2008) were among the first to use Naive Bayes and Decision Tree methods to generate two types of models that classify students' self-efficacy as high or low. Their data, derived from 33 students using an intelligent tutoring system, was based on demographics for the first model and added pre-test scores, physiological data, and student behaviors for the second. On the other hand, Ezen-Can and Boyer (2014) used a k-medoids clustering algorithm, focusing on student dialogue, and found gender and self-efficacy affected utterance confidence. Furthermore, Rizk and Farooque (2021) assessed computer science students' self-efficacy through a factor analysis survey, ranking problem-solving as the foremost determinant of self-efficacy among computer science students. Lastly, Tan and Cutumisu (2022) employed Random Forest and XGBoost algorithms on a massive dataset from the PISA 2018, highlighting non-cognitive factors, like life meaning and task mastery motivation, as primary self-efficacy influencers. Notably, XGBoost slightly surpassed Random Forest in prediction precision.

In the context of Muslim societies, there is a lack of empirical studies on educational and human development as well as a dearth of data-driven modeling studies predicting students' self-efficacy (Nasser et al., 2019). Thus, the IIIT, a non-profit organization that seeks to provide a platform for the unique perspectives of Muslim thinkers, scholars, and practitioners in the humanities and social sciences, launched an initiative in 2018 called Advancing Education in Muslim Societies (AEMS) to address this gap. This initiative aims to investigate the knowledge, abilities, and attitudes that can enhance educational opportunities and human development in Muslim societies.

Numerous studies have examined education programs related to employment and citizenship, but almost none have considered the social and emotional characteristics of Muslim societies (Nasser et al., 2019). The AEMS initiative recognizes the importance of not overemphasizing East-West, secular-religious, or North-South boundaries on epistemological and methodological understandings in an era of globalization and multigenerational immigrant communities. However, many external human development and education reform initiatives aimed at the Muslim world have been rooted in secular, individualist values. As these measures are implemented in religious, collectivist, and community-focused societies, their capacity to serve the needs of local populations is diminished (Davies, 2016; McKenzie, 2012). Thus, AEMS selects constructs important to socio-emotional education (empathy, forgiveness, moral reasoning, and community-mindedness) that are universal, foundational to Islamic values, and considered high value in many Muslim communities when framed in terms of spiritual and moral growth (Nasser et al., 2019).

## 3 Method

This study utilized a structured quantitative approach to address the research questions stated in the introduction. The first step involved retrieving the data, which was followed by data exploration. Data exploration is an essential preliminary investigation of the dataset to gain a better understanding of it. To make optimal use of the available information, the study involved multiple steps, including data cleaning, selecting relevant variables, reverse coding, screening for missing values, and grouping relevant items. These steps are described in detail in the following sections, outlining the procedures taken for the exploration analysis.

Once the exploration phase was completed, different machine learning models, including Decision Tree, Random Forest, XGBoost, and Neural Network, were employed and evaluated to address the research questions. The study utilized evaluation criteria to assess the models' efficiency and accuracy. The most common performance metrics for regression model evaluations are root mean square error (RMSE) and *R*^2^ (Suha and Sanam, 2022). Therefore, the outcome performance metrics for each model were captured and reported. Before running the models, the dataset was split into two subsets: a training set (80%) and a testing set (20%).

**Figure 1:**
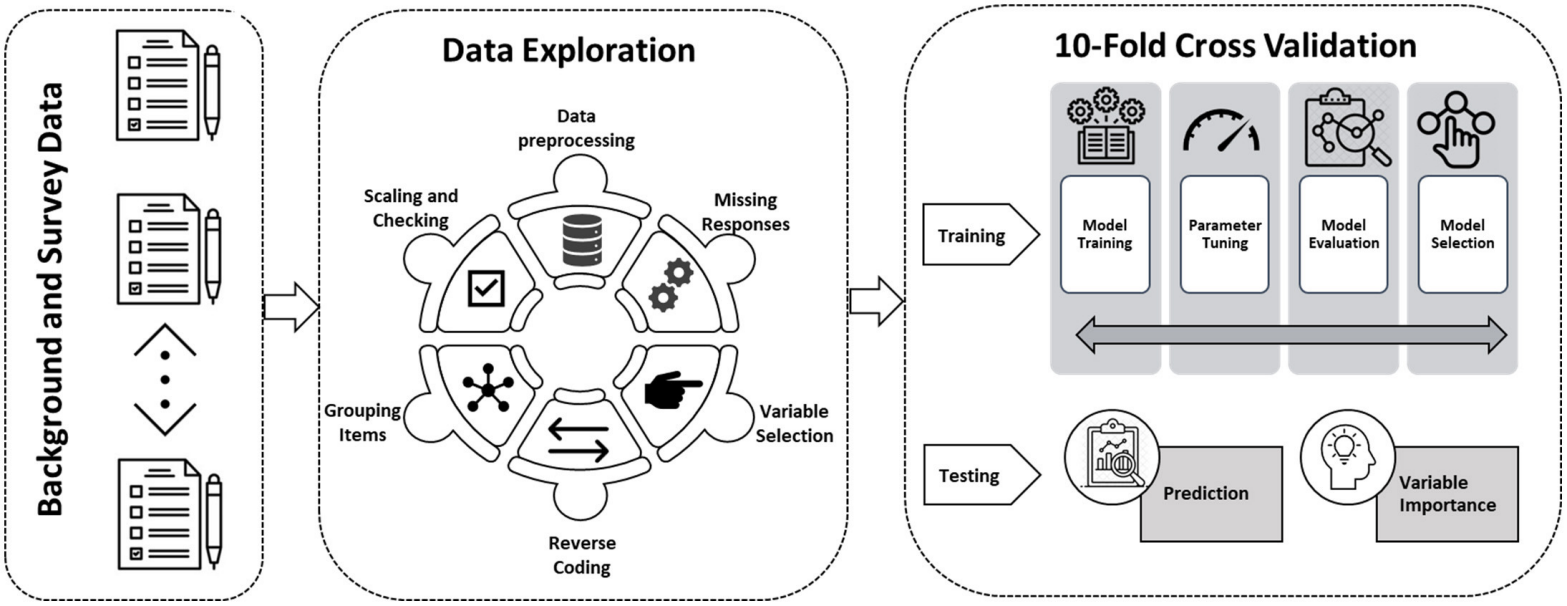
Machine learning framework used in this study.

To summarize the steps taken in this study, a framework is presented in Figure 1. This framework delineates the various stages, spanning from data retrieval and modeling development to model evaluation and reporting the results. The study aimed to provide a detailed and structured approach to answer the research questions in a rigorous and systematic manner, utilizing various techniques to optimize the performance of the machine learning models.

### 3.1 Data collection

The primary data source was an empirical survey from the IIIT. The IIIT conducted two waves of its “Mapping the Terrain” survey in 2018–2019 and 2019–2020, asking K-12 and university students and teachers in Muslim-majority societies to respond to a variety of prompts, each focused on particular values and competencies. The survey included constructs measuring a subset of socio-emotional traits (e.g., empathy), cognitive traits (e.g., problem-solving), and other factors relevant to Muslim societies within a human development trajectory (see Tawil and Cougoureux, 2013).

This study employed data collected in the 2019–2020 Mapping the Terrain survey, which reached ~20,000 participants from 15 countries. The survey explored the values and competencies in Muslim societies with a focus on secondary school and university students and their instructors. The variables, focused on students, are defined in Table 2, and adopted from Nasser and Saroughi (2021).

**Table 2:** Variable definitions.

**Factor (Abbreviation)**	**Definition**
Self-efficacy (SEFF)	A person's belief in his or her ability to organize and execute certain behaviors that are necessary to become successful in each task
Collectivist vs. individualist orientation (CIO)	Collectivism is a situation in which people feel they belong to larger in-groups, while individualism is a situation in which people are concerned with themselves and close family members only
Forgiveness (FORG)	The ability and willingness to let go of hard feelings and the need to seek revenge on someone who has wronged the subject or committed a perceived injustice against the subject or others
Problem-solving (PSOLV)	Skills that individuals use to analyze, understand, and prepare to respond to everyday problems, decisions, and conflicts
Sense of belonging (SOB)	An individual's feeling of identification with a certain group
Religiosity/spirituality (RELSPIR)	The degree of influence one's faith has on one's values, behaviors, and everyday life
Gratitude (GRAT)	The appreciation of what is valuable and meaningful to oneself and represents a general state of thankfulness and/or appreciation
Self-regulation (SREG)	Generated thoughts, feelings, and actions that are planned and cyclically adapted to the attainment of personal goals
Meaning-making (MEANMK)	Sense of coherence or understanding of existence, a sense of purpose in one's life, the pursuit and attainment of worthwhile goals, and an accompanying sense of fulfillment
Empathy (EMP)	The ability to understand others' emotion, the willingness to care, feel, and take the perspective of others and be responsive to their needs
Emotion regulation (EMOREG)	A process through which individuals modulate their emotions consciously and non-consciously to respond appropriately to environmental demands

Self-efficacy was the target/predicted variable in this study, while the rest were predictor variables.

The survey was distributed to four groups (secondary school students, university students, secondary school teachers, and university instructors). The present study focused on secondary school students to understand the factors most strongly predicting self-efficacy in that group. Furthermore, these students represented the majority of survey respondents (further described in the descriptive analysis section). Respondents were also restricted to the main region of each participating country due to regional differences, financial considerations, host-country approval, and location of affiliate offices (Nasser and Saroughi, 2021). However, randomization was performed on the classroom level to provide every participant a chance to be considered in the survey (Nasser and Saroughi, 2021).

### 3.2 Data description

As reported by the IIIT, the total responses per group in the original dataset were as follows: 11,391 secondary school students, 4,698 university students, 2,218 school teachers, and 593 university instructors. Furthermore, each factor (described in Table 1) was measured using a different number of items, ranging from a minimum of five to a maximum of 18. The number of items for each factor was as follows: forgiveness (9 items), collectivist vs. individualist orientation (14), self-efficacy (12), problem-solving (12), sense of belonging (18), religiosity/spirituality (5), gratitude (6), self-regulation (16), meaning-making (10), empathy (7), and emotion regulation (8). Each item was measured on a 4-point Likert scale ranging from lowest to highest in ascending order. Since the present study focused on school students who participated in the survey, not all questions were relevant. Therefore, only questions (i.e., survey items) answered by secondary school students were considered in this study.

### 3.3 Data selection

Retrieving the dataset of interest began by selecting the responses of secondary school students. As noted above, 11,391 were identified as the initial sample. Based on this selection, it was observed that all the selected factors (the 11 factors described in Table 1 for this study) were answered by these students. However, based on the report issued by the IIIT for the 2019–2020 survey, some of the demographic variables were only answered by other participants (university students, secondary school teachers, and university instructors). These variables were related to work experience, number of children, relationship status, degree year, and education level. Due to the focus of this study on secondary school students, these five demographic variables were removed. Also, there were 4,323 missing values among secondary school students' observations, which were eliminated based on stakeholder recommendations. Furthermore, 6,424 of the secondary school students included were between 15 and 18 years old, 619 were between 18 and 24, and 25 were over 24. However, students generally conclude their secondary school education at or around age 18 (National Center for Education Statistics, 1996). Thus, this study only selected responses from students under 18 based on that estimate and stakeholder recommendations. After missing values and non-relevant observations were eliminated, the final sample size used for further analysis was 6,424.

The final variables consisted of 10 factors measured using 117 items and two demographic variables for students under 18. Further analysis was conducted by reversing 35 items in the survey because of how they were phrased. The two demographic variables were gender (male or female) and country (referred to as region in this study): ungrouped, Southeast Asia, Central Asia, Middle East and North Africa (MENA), Sub-Saharan Africa, and South Asia. Table 2 depicts the final set of variables, including the factors and their corresponding number of items and the categorical variables with their levels.

### 3.4 Descriptive analysis

In order to make the most of the data, it was essential to study the properties of the variables by utilizing summary statistics and visualization techniques. Using these methods, it was important to identify correlations, trends, and outliers that might have influenced the analysis.

The descriptive statistics for student self-efficacy are reported in Table 3. Self-efficacy was the dependent variable and was measured using 12 items. The 12 items were summed and then averaged per participant to compute the final value for each participant. A similar procedure was conducted with the independent variables.

**Table 3:** Descriptive statistics for variables used in the study (*N* = 6,424).

**Continuous variable**	**Survey items**	**Mean (*SD*)**	**Minimum**	**Maximum**	**Cronbach's alpha**
Forgiveness (FORG)	9	2.33 (0.56)	1	4	0.75
Collectivist vs. individualist orientation (CIO)	14	2.68 (0.29)	1	3.78	0.65
Self-efficacy (SEFF)	12	3.03 (0.41)	1.17	4	0.66
Problem-solving (PSOLV)	12	3.13 (0.47)	1	4	0.79
Sense of belonging (SOB)	18	2.98 (0.43)	1	4	0.81
Religiosity/spirituality (RELSPIR)	5	3.44 (0.40)	1	4	0.87
Gratitude (GRAT)	6	3.12 (0.44)	1.33	4	0.57
Self-regulation (SREG)	16	2.87 (0.42)	1.31	4	0.87
Meaning-making (MEANMK)	10	3.03 (0.44)	1	4	0.69
Empathy (EMP)	7	2.93 (0.39)	1.14	4	0.60
Emotion regulation (EMOREG)	8	3.03 (0.46)	1	4	0.64
**Categorical variable (levels)**		**Frequency**		**Percent**	
**Region (6)**					
Ungrouped		673		10.48%	
Southeast Asia		959		14.93%	
Central Asia		1,115		17.36%	
MENA		416		6.48%	
Sub-Saharan Africa		837		13.03%	
South Asia		2,424		37.73%	
**Gender (2)**					
Male		2,497		38.87%	
Female		3,927		61.13%	

Self-efficacy is a continuous variable representing secondary school students under the age of 18, ranging from a minimum of 1 to a maximum of 4, with a mean of 3.03 (*SD* = 0.41). The majority of the independent variables described in Table 3 (FORG, PSOLV, SOB, RELSPIR, GRAT, MEANMK, EMP, and EMOREG) had means of ~3 or above. CIO and SREG had the lowest means: 2.68 and 2.87, respectively.

Furthermore, the reliability of each variable (see Table 3) was evaluated to ensure that items corresponding to each variable sufficiently reflected the scope of each variable. Reliability measures the degree of consistency between multiple measurements or items for a single variable (Hair, 2010). The reliability measure (i.e., Cronbach's alpha) ranges between 0 and 1, in which 0.60–0.70 is the commonly accepted level (Hair, 2010).

All variables included in the study had a Cronbach's alpha of 0.6 or above, except gratitude (0.57). However, Cronbach's alpha is sensitive to the number of items (the more items, the higher the Cronbach's alpha score). Because gratitude was very close to 0.60 and was among the lowest variables in terms of the number of items, it was kept in this study to investigate its role in self-efficacy and derive more insightful conclusions. The last two demographic variables were region and gender. Region was a categorical variable consisting of six levels: ungrouped (with a frequency of 673 students or 10.48% out of 6,424) representing the U.S. and Bosnia, Southeast Asia (959 students or 14.93%), Central Asia (1,115, 17.36%), MENA (416, 6.48%), Sub-Saharan Africa (837, 13.03%), and South Asia (2,424, 37.73%). As shown in Table 2, the most common region in this study was South Asia (representing 37.73% of responses), whereas MENA had the lowest response rate (6.48%).

**Figure 2:**
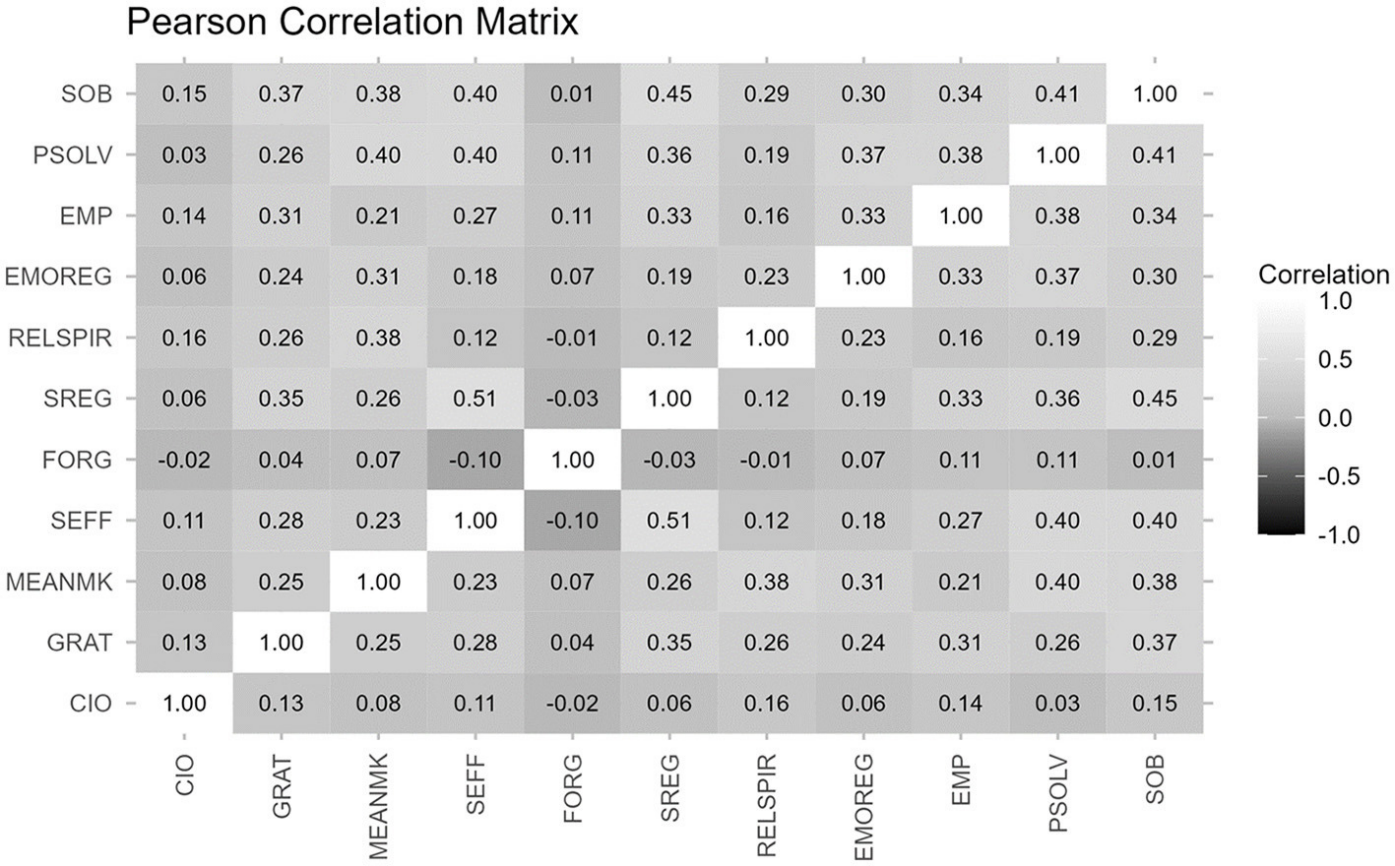
Correlations between variables.

To gain a better understanding of the relationship between the variables, Pearson correlation was conducted, as depicted in Figure 2. The overall correlation between variables appeared to be low to moderately correlated. Given that the data were collected through a survey, multicollinearity may be present, which could have an impact on the algorithm's performance. However, this study also utilized tree-based models, which are robust to multicollinearity due to their random nature (Al-Nammari et al., 2023).

**Figure 3:**
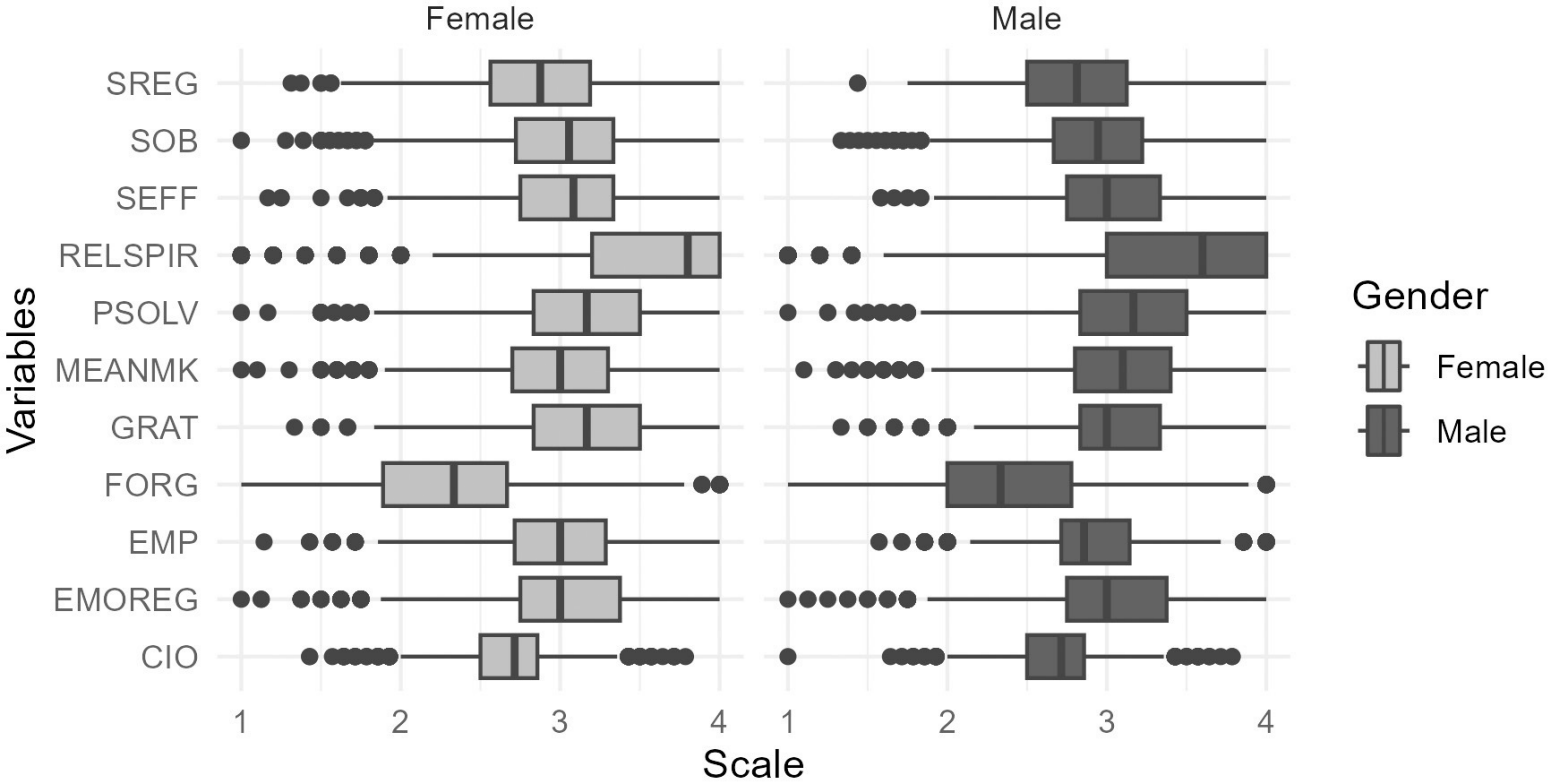
Distribution among variables based on gender.

A boxplot was used to understand the distribution of the factors based on gender, highlighting differences in central tendencies and variability between male and female participants (see Figure 3). It suggests that, while many factors showed comparable distributions across gender, women tended to have higher medians for certain factors (e.g., SOB, GRAT, and SEFF). The variability in the data offers further insight into the range of responses within each gender group.

**Figure 4:**
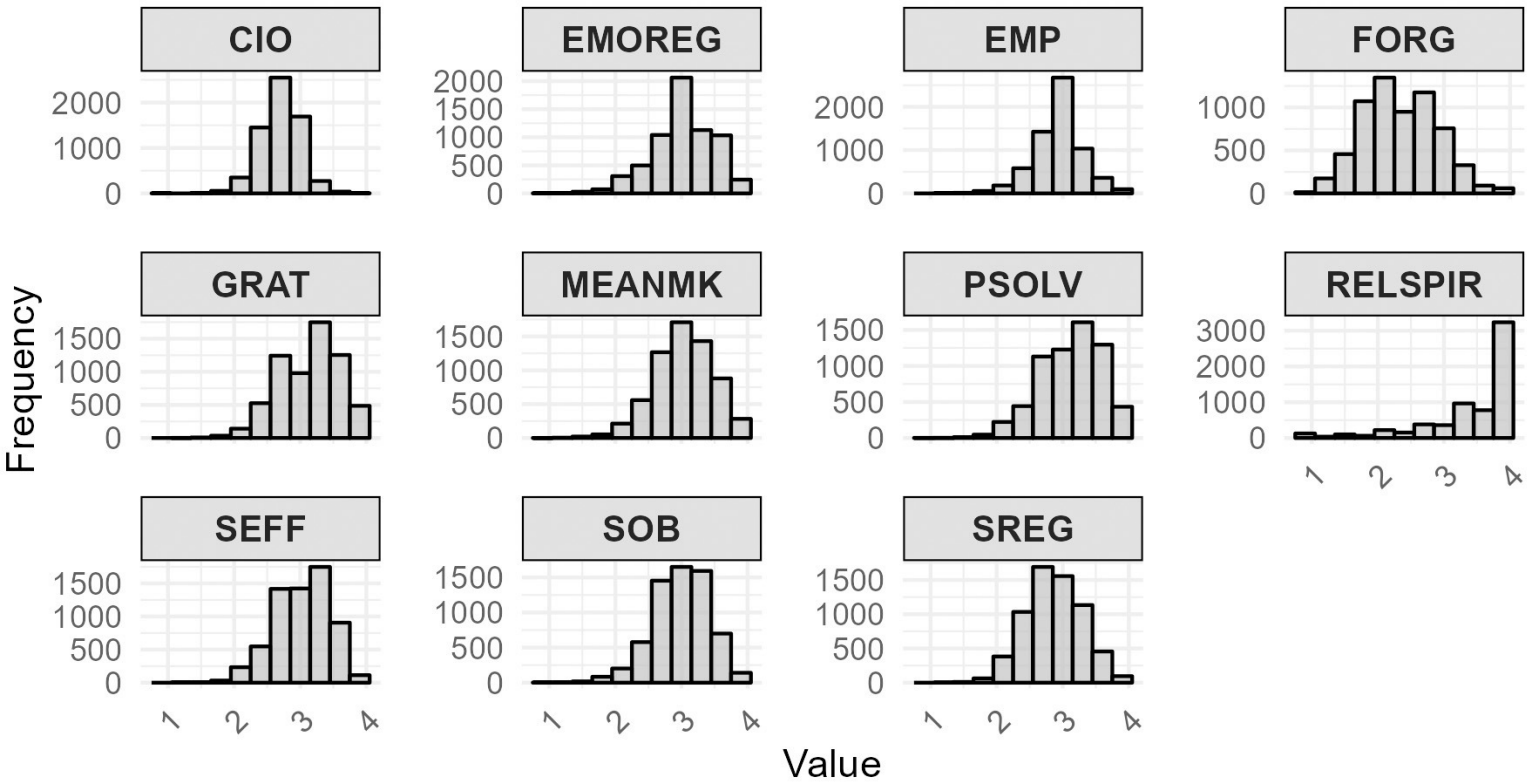
Frequency per variable.

To examine the frequency distribution of responses for each factor, a histogram was developed for each factor (see Figure 4). The histograms reveal that some of the constructs, such as SEFF, EMP, and MEANMK, exhibited a moderate score of around 3, indicating a generally balanced perception among participants. Constructs like RELSPIR and EMOREG were right-skewed, suggesting stronger ratings, with many participants reporting high levels in these areas. Conversely, constructs like FORG showed a slight left-skew, indicating lower ratings by some participants. The variety in distribution shapes highlights consistent patterns across some constructs, while others display more variability, reflecting diverse perceptions and tendencies within the sample.

### 3.5 Machine learning models

Machine learning has been successfully employed across various sectors, including healthcare (Araz et al., 2019), employment of people with disabilities (Sobnath et al., 2020), and transportation (Oliveira Almeida et al., 2022). In education research, machine learning offers significant promise in revealing impactful patterns that can enhance educational outcomes (Peña-Ayala, 2014). There is mounting scholarly interest in harnessing machine learning to explore educational challenges, such as assessing student performance (Asselman et al., 2021), understanding dropout rates (Devi and Ratnoo, 2022), and analyzing student behaviors (Hooshyar et al., 2019).

This study sought to predict the self-efficacy of secondary school students in Muslim societies through supervised machine learning algorithms. Depending on the specific outcome in focus, these algorithms can address either classification or regression challenges (Simsekler et al., 2021b). Their efficacy in tackling diverse problem sets—be it regression or classification—has been validated, along with their capacity to discern variable significance (Simsekler et al., 2021b). Given that the study's outcome, self-efficacy, was a continuous variable, regression-based models were employed.

To predict student self-efficacy in public schools within Muslim societies, four machine learning models were used: Decision Tree (bagging), Random Forest, XGBoost, and Neural Network (James et al., 2013). Random Forest, initially developed by Breiman (2001), is a non-parametric method that has received attention for its flexibility in handling regression and classification problems (Simsekler et al., 2021a). Random Forest is a type of ensemble learning algorithm that combines many decision trees based on a random selection of variables. More specifically, this means that multiple decision trees are created by randomly selecting a subset of variables from the original dataset (Tan and Cutumisu, 2022). Random Forest can quickly and effectively handle a large number of input variables without overfitting, providing accurate predictions (Liu et al., 2021). Decision Tree (bagging or bootstrap aggregation) is another derivative of decision tree and ensemble learning methods. This algorithm creates multiple trees by selecting a sample of the data with replacement. XGBoost is also an ensemble machine learning algorithm built on decision tree models (Chen and Guestrin, 2016). Deep Neural Network comprises an input layer, hidden layers, and an output layer and utilizes multiple layers of neurons to process information. By adjusting the weights of the network, deep neural networks are trained to accurately predict outcomes based on the input data (Ljubic et al., 2022).

It is common practice in predictive modeling studies, especially those focusing on self-efficacy and behavioral predictions, to apply multiple similar algorithms to determine which offers the best performance for the dataset. Although Decision Tree, Random Forest, and XGBoost are all tree-based methods, each has distinct mechanisms that can result in different predictive accuracies. For instance, Random Forest uses bagging to enhance variance reduction, while XGBoost applies boosting techniques to reduce bias, making these methods complementary rather than redundant. This approach was consistent with prior studies in related fields, where similar models have been tested to identify the most accurate one (Benbelkacem et al., 2019; Tan and Cutumisu, 2022). Linear regression was avoided in this study due to its assumption of a linear relationship between the response and predictors, which is not always applicable for educational and psychological datasets that may exhibit complex, nonlinear relationships. In contrast, the tree-based models and neural networks applied here do not require such assumptions, making them more appropriate for capturing the interactions among predictors in our dataset (Benbelkacem et al., 2019).

This study utilized a dataset of 6,424 responses and 12 predictors for the predictive models. In machine learning, the amount of data required is influenced by model complexity, data dimensionality, the field of application, and the generalization objectives of the study. While deep learning models benefit from large datasets, structured data with moderate dimensionality (such as survey responses with 12 predictors) can be effectively modeled with smaller datasets, especially with ensemble methods like Random Forest and XGBoost, which perform robustly on smaller datasets due to their bagging and boosting mechanisms (Breiman, 2001; Chen and Guestrin, 2016). These ensemble models are designed to minimize variance and enhance predictive accuracy, capturing complex interactions among predictors without the need for extensive datasets (James et al., 2013). Additionally, using a sample size in the range of several thousand responses aligned with common practices in educational and behavioral sciences, where models on similar data scales have been successfully built for predictive tasks related to self-efficacy and related constructs (McQuiggan et al., 2008). Prior research has successfully employed machine learning models (e.g., Neural Networks and Random Forest) with much smaller datasets, such as Sahlaoui et al. (2021), which utilized fewer than 300 observations, demonstrating the viability of deep learning approaches in similar contexts.

## 4 Results

**Figure 5:**
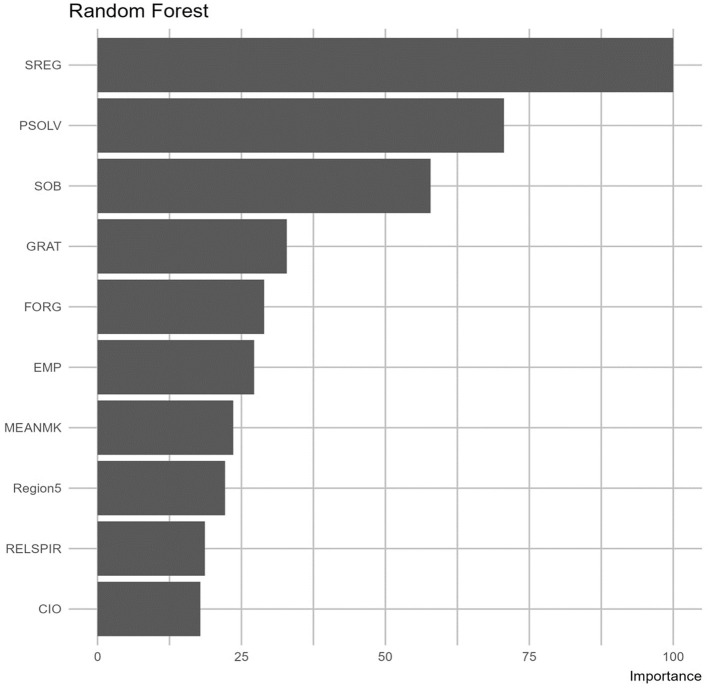
Importance of variables based on random forest.

**Figure 6:**
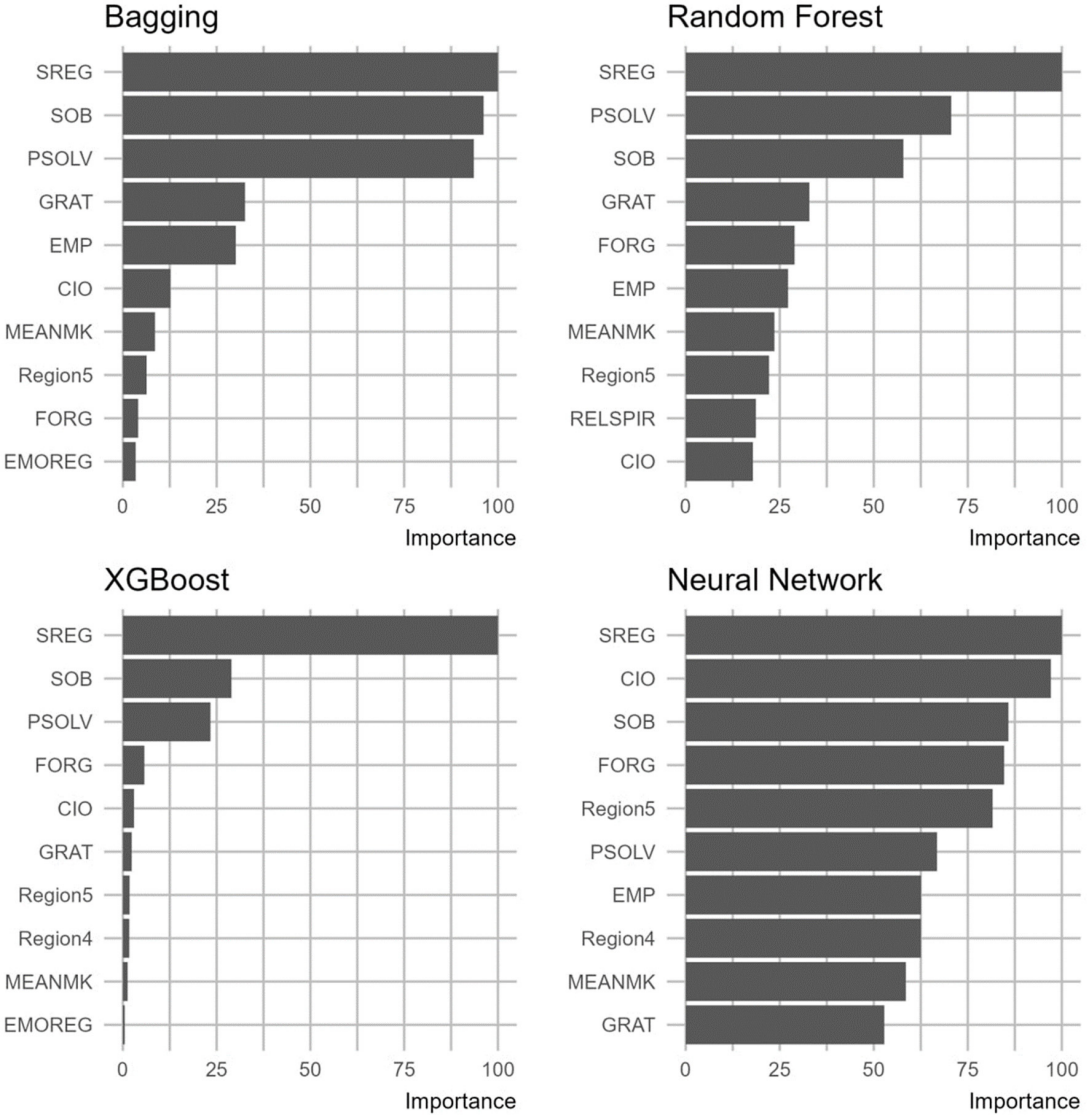
Importance of variables based on the four machine learning algorithms.

Figure 5 illustrates the significance of the top 10 variables for predicting self-efficacy within the target demographic. The importance of each variable is gauged by examining how the removal of that particular variable impacts the model's performance; more specifically, its absence or presence would cause a change to the model's performance metrics proportionally to the variable's designated importance. From the findings, self-regulation (SREG), problem-solving (PSOLV), and sense of belonging (SOB) emerged as the most important variables in both the Decision Tree and Random Forest models. XGBoost, to some extent, concurred with these models, identifying self-regulation (SREG) as the paramount variable. Figure 6 shows the variable importance yielded from each model.

The performance metric of each model is reported in Table 4. Based on the results, Random Forest and Neural Network outperformed the other models in terms of the evaluation metrics *R*^2^ and RMSE. However, Random Forest demonstrated the most consistent performance between training and testing data, with *R*^2^ scores of 38.61 and 38.91, and RMSE values of 32.29 and 32.67. This consistency indicates that the model did not overfit, making it the best-performing model overall. In the Random Forest model, self-regulation appeared to be the most powerful predictor with a relative importance of 100%, followed by problem-solving (80%), sense of belonging (63%), gratitude (40%), forgiveness (35%), empathy (28%), meaning-making (25%), and religion/spirituality (20%).

**Table 4:** Performance metrics for machine learning models.

**Predictive model**	**Training**		**Testing**	
	*R* ^2^	**RMSE**	*R* ^2^	**RMSE**
Decision tree (bagging)	30.66	34.11	31.07	34.46
Random forest	38.61	32.29	38.91	32.67
XGBoost	35.01	33.07	36.61	33.17
Neural network	36.22	32.67	39.06	32.31

## 5 Discussion

### 5.1 Key findings

Random Forest was chosen for the discussion because it outperformed the other models with respect to the evaluation metrics *R*^2^ (38.91%) and RMSE (32.67). Random Forest is often highly effective on datasets with moderate size and lower dimensionality, such as ours, as it reduces variance through bagging, which helps prevent overfitting (Breiman, 2001). In contrast, while XGBoost has advantages in handling larger, high-dimensional datasets and dealing with complex interactions, its additional regularization and boosting iterations did not improve accuracy as effectively in this context. XGBoost's training complexity can sometimes lead to overfitting, especially when there is a limited number of features and observations (Chen and Guestrin, 2016). Therefore, Random Forest's simplicity and robustness given the dataset's size and structure likely contributed to its superior performance for this predictive task. The primary technical challenge of Random Forest was ensuring its ability to handle the complex interactions between socio-emotional and cultural predictors within a moderately sized dataset. The model's strength lies in its ensemble approach, which combines multiple decision trees to capture nuanced patterns without overfitting—a common risk with high-dimensional data (Breiman, 2001). Additionally, the decision to select Random Forest over more complex models like Neural Networks or XGBoost was guided by the model's robustness in terms of multicollinearity and its interpretability. This robustness allowed for a clearer assessment of feature importance, which was crucial in a study aimed at identifying key predictors of self-efficacy in a novel educational context. Overall, the technical challenge was to configure Random Forest in a way that would maximize predictive accuracy while retaining interpretability, which is essential for informing educational strategies based on the model's insights.

Interpretation of an *R*^2^ value depends on the purpose of the research and the application domain. When compared to studies in pure technical fields (e.g., engineering), modeling studies in social and behavioral science typically report low *R*^2^ values. Cohen (1988) established a rule for interpreting *R*^2^ in behavioral and social science, stating that a model with an *R*^2^ >0.26 is considered substantial. In this study, the *R*^2^ was around 39%, which would be considered adequate and sufficient for the research purpose. The three constructs that showed the highest importance (above 50%) were self-regulation, problem-solving, and sense of belonging. The next four showed moderate importance (between 50% and 25%): gratitude, forgiveness, empathy, and meaning-making. The last two in the top predictor list showed lower importance (below 25%): region and religion/spirituality. Finally, there were three variables not included in the top predictors: gender, emotion regulation, and collectivist vs. individualist orientation.

The results of the Random Forest model showed that the most important predictor of self-efficacy was self-regulation. This result was the same across all models tested. The strong link between self-efficacy and self-regulation aligned with theories and other empirical findings in academic settings. Self-regulation involves the proactive direction of one's behavior to attain self-set goals. When unable to achieve their goals initially, learners rely on affective, cognitive, motivational, and behavioral feedback to alter or adjust their tactics and behavior (Zimmerman, 1989). According to Bandura (1986), self-regulation and self-efficacy are distinct but interrelated and mutually reinforcing concepts. He argued that successful experiences with self-regulation can increase a person's self-efficacy, as they learn to trust in their ability to regulate their behavior and achieve their goals. Bandura also argued that self-efficacy is a key component of self-regulation, as it provides the motivation and confidence necessary to regulate one's thoughts, emotions, and behaviors. An empirical study of university students found that those who reported better levels of self-regulation were more inclined to believe in their academic abilities (Wolters and Hussain, 2015). These findings emphasize the significance of establishing self-regulation abilities to increase self-efficacy and success in academics and other areas of life. Self-regulation can be learned through modeling (English and Kitsantas, 2013; Schunk, 2005).

The second strongest predictor of self-efficacy was problem-solving, which refers to the skills people employ to analyze, comprehend, and prepare their responses to everyday problems, decisions, and confrontations. It is a cognitive-behavioral process in which people seek to solve real-world problems in a social setting (Siu and Shek, 2010). Several studies have identified positive relationships between self-efficacy and problem-solving (Bandura, 1993, 1997; Erozkan, 2014). Erozkan (2014) found that an increase in constructive problem-solving and the insistent-persistent approach was linked to an increase in social self-efficacy. On the other hand, as social problem-solving decreased, so did social self-efficacy. The study concluded that social problem-solving skills were an essential predictor of self-efficacy.

The third strongest predictor of self-efficacy was sense of belonging. A student's sense of belonging in an academic institution is defined as their perception that they have been supported, accepted, respected, and included in the institution (Nasser and Saroughi, 2021). Research has widely confirmed the positive effects of a general sense of belonging on a variety of individual physical, psychological, and social outcomes (e.g., Slaten et al., 2016). Studies have shown that positive school environments can lead to positive outcomes for students (e.g., Church et al., 2001; Roeser et al., 1996) and that students' experience of acceptance influences multiple dimensions of their behavior and attitudes (Battistich et al., 1995; Osterman, 2000). One way that sense of belonging could affect self-efficacy is through interpersonal relationships with teachers. Students can develop attachments to their teachers and see them as role models; this can influence how they interpret their vicarious experiences, which can then positively or negatively impact their confidence, motivation to learn, and self-efficacy beliefs (Bandura, 1997; Ryan et al., 1994).

The present study found that gratitude, forgiveness, empathy, and meaning-making were moderately strong predictors of general self-efficacy, with the relationships ranging below 50% and above 25%. Bandura (1977) social cognitive theory, which emphasizes the influence of psychological and emotional states on self-efficacy, may explain this relationship. The results support previous research in which gratitude had a significant association with self-efficacy and played a vital role in positive functioning and wellbeing (e.g., Emmons and McCullough, 2003; Froh et al., 2008; Rey, 2009). Expressing gratitude has also been shown to increase prosocial behaviors and coping strategies (Ting and Yeh, 2014). Additionally, forgiveness (Baghel and Pradhan, 2014; Ferudun and Onur, 2018), empathy (Baghel and Pradhan, 2014; Schurz, 2018), and meaning-making (DeWitz et al., 2009) have been linked to self-efficacy. Overall, these findings support previous studies that found a positive relationship between self-efficacy and positive psychological variables.

The last two of the top 10 predictors (region and religion/spirituality) showed a predictive power lower than 25%. Previous studies have demonstrated the importance of spirituality in predicting self-efficacy (Rakhshanderou et al., 2021). The demographic variable of region could reflect regional differences in socioeconomic factors, which could affect self-efficacy, as shown in previous studies (Boardman and Robert, 2000). Regional or national socioeconomic factors may have an impact on perceived self-efficacy beyond individual-level socioeconomic status for several reasons. For instance, limited growth opportunities in resource-constrained areas may influence an individual's belief in their ability to achieve success, and the broader social context of an area could also influence self-efficacy, as individuals may be exposed to differing levels of vicarious mastery experiences depending on the socioeconomic status of those around them.

Three variables (gender, emotion regulation, and collectivist vs. individualist orientation) were not among the top 10 predictors of self-efficacy in the Random Forest model. The result for gender was consistent with a previous meta-analysis that found only a minor difference in academic self-efficacy based on gender (Huang, 2013). However, previous research from specific countries has found a significant gender difference in terms of academic self-efficacy (Wang et al., 2019). The influence of gender on self-efficacy could be a result of the self-beliefs that underlie those differences. Studies have indicated that some gender differences in academic, social, and personality aspects could be attributed to gender orientation, which refers to the stereotypical beliefs about gender that individuals hold, rather than biological sex (Usher and Pajares, 2008). In other words, the traditional beliefs and societal expectations that people have about what men and women should be good at may impact their confidence and abilities in different domains. The finding that emotion regulation was not shown to be an important predictor of self-efficacy supported the suggestion by Bong and Clark (1999) that self-efficacy is mainly influenced by cognitive judgment but does not necessarily negate the role of emotional experience in influencing self-efficacy, as suggested by Bandura (1977). Some empirical studies have established a significant association between emotion regulation and self-efficacy, but it is still unclear how emotion regulation strategies contribute to the development of self-efficacy (Deng et al., 2022; Lande et al., 2023). Lastly, a collectivist vs. individualist cultural orientation did not show significant predictive power in the model.

### 5.2 Implications

There is a lack of empirical studies on educational and human development in Muslim contexts, particularly those employing data-driven modeling to predict students' self-efficacy. To address that gap, this study presents data-driven insights into predictors of secondary school students' self-efficacy in Muslim-majority societies, leveraging primary data sourced from the IIIT's Mapping the Terrain survey. The research emphasizes the integration of socio-emotional and cognitive traits that are often sidelined in mainstream research and constructs but are deeply embedded in many Muslim societies, such as the significance of community, empathy, and moral reasoning. This investigation fills a notable gap in global educational discourse, emphasizing socio-emotional dimensions of education pertinent to Muslim contexts, thereby guiding international and local stakeholders toward more contextually apt educational strategies.

The research also ranked the relative importance of self-efficacy predictors, which can aid in operationalizing self-efficacy and prioritizing interventions. The resulting predictive model could assist educators in identifying students with relatively lower or higher levels of self-efficacy and developing interventions and strategies to improve their self-efficacy and academic achievement. It is noteworthy that all factors that were highly or moderately important in predicting self-efficacy are teachable or can be improved via evidence-based interventions. For example, self-regulation skills can be learned through modeling as well as specific learning environment features and teaching practices (English and Kitsantas, 2013; Schunk, 2005). Also, researchers have identified evidence-based practices to enhance social problem-solving skills among students through a combination of cognitive and behavioral techniques (Merrill et al., 2017; Smith and Daunic, 2006; Gootman, 2001). Furthermore, enhancing students' sense of belonging in schools is feasible, and its importance is underscored by substantial research. A meta-analysis spanning 51 studies pinpointed teacher support as a pivotal factor in fostering students' feelings of school affiliation (Allen et al., 2018).

### 5.3 Conclusion

This study employed machine learning techniques to predict student self-efficacy in Muslim societies, incorporating a comprehensive set of factors. The results revealed that self-regulation, problem-solving, and sense of belonging were the top three influential predictors of student self-efficacy, surpassing the 50% threshold. Conversely, gender, emotion regulation, and collectivist vs. individualist orientation did not emerge as significant predictors. Moderate importance (25–50%) was observed for four constructs: gratitude, forgiveness, empathy, and meaning-making, while region and religion/spirituality exhibited limited predictive power (< 25%).

The study's findings could contribute to the development of interventions and strategies that enhance student self-efficacy and academic achievement, ultimately improving the overall quality of the education system. Policymakers could use these findings to create programs that foster self-regulation, problem-solving, and a sense of belonging among students. The findings could also enrich theoretical models of self-efficacy and help identify students with relatively lower or higher levels of self-efficacy. Additionally, using machine learning to predict student self-efficacy in a new context and using a new dataset expands the application of machine learning algorithms and highlights their potential in educational research.

Future research could build on and address this study's limitations. The study focused on 10 continuous factors and two categorical/demographic variables, but other important factors may influence student self-efficacy, such as family support and socioeconomic status. Future research could consider incorporating these factors to improve the predictive power of the model, and more tuning techniques could be used to improve the model's performance. The study used a cross-sectional design, which does not allow for causal inferences or the examination of changes in self-efficacy over time. A longitudinal design would be needed to better understand the trajectory of self-efficacy and how it is influenced by various factors. In addition, self-efficacy belief could be examined over time using system dynamics simulations to test various hypotheses. Finally, this study relied on self-reported data, which may be subject to social desirability bias or other biases. In future research, alternative techniques may be employed to collect real-time, objective measures for predicting student self-efficacy.

## Data Availability

The original contributions presented in the study are included in the article/supplementary material, further inquiries can be directed to the corresponding author.

## References

[B1] Abu-Nimer M. Nasser I. (2017). Building peace education in the Islamic educational context. Int. Rev. Educ. 63, 153–167. 10.1007/s11159-017-9632-7

[B2] Allen K. Kern M. L. Vella-Brodrick D. Hattie J. Waters L. (2018). What schools need to know about fostering school belonging: a meta-analysis. Educ. Psychol. Rev. 30, 1–34. 10.1007/s10648-016-9389-8

[B3] Al-Nammari R. Simsekler M. C. E. Gabor A. E. Qazi A. (2023). Exploring drivers of staff engagement in healthcare organizations using tree-based machine learning algorithms. IEEE Trans. Eng. Manag. 70, 2988–2997. 10.1109/TEM.2022.3209879

[B4] Araz O. M. Olson D. Ramirez-Nafarrate A. (2019). Predictive analytics for hospital admissions from the emergency department using triage information. Int. J. Prod. Econ. 208, 199–207. 10.1016/j.ijpe.2018.11.024

[B5] Asselman A. Khaldi M. Aammou S. (2021). Enhancing the prediction of student performance based on the machine learning XGBoost algorithm. Interact. Learn. Environ. 31, 3360–3379. 10.1080/10494820.2021.1928235

[B6] Baghel S. Pradhan M. (2014). Self-efficacy as a moderator between empathy and forgiveness relationship. Indian J. Pos. Psychol. 5, 388–392. 10.15614/ijpp/2014/v5i4/88461

[B7] Bandura A. (1977). Self-efficacy: toward a unifying theory of behavioral change. Psychol. Rev. 84, 191–215. 10.1037/0033-295X.84.2.191 847061

[B8] Bandura A. (1986). Social Foundations of Thought and Action. Englewood Cliffs: Prentice Hall.

[B9] Bandura A. (1993). Perceived self-efficacy in cognitive development and functioning. Educ. Psychol. 28, 117–148. 10.1207/s15326985ep2802_3

[B10] Bandura A. (1997). Self-Efficacy: The Exercise of Control. New York: W.H. Freeman and Company.

[B11] Bandura A. (2006). “Guide for constructing self-efficacy scales,” in Self-Efficacy Beliefs of Adolescents, eds. F. Pajares and T. Urdan (Greenwich, CT: Information Age Publishing), 307–337.

[B12] Bandura A. (2018). Toward a psychology of human agency: pathways and reflections. Perspect. Psychol. Sci. 13, 130–136. 10.1177/1745691617699280 29592657

[B13] Bandura A. Barab P. G. (1973). Processes governing disinhibitory effects through symbolic modeling. J. Abnorm. Psychol. 82, 1–9. 10.1037/h0034968 4738326

[B14] Battistich V. Solomon D. Kim D. Watson M. Schaps E. (1995). Schools as communities, poverty levels of student populations, and students' attitudes, motives, and performance: a multilevel analysis. Am. Educ. Res. J. 32, 627–658. 10.3102/0002831203200362738293548

[B15] Benbelkacem S. Kadri F. Atmani B. (2019). Machine learning for emergency department management. IJISSS. 11, 1–20. 10.4018/IJISSS.2019070102

[B16] Boardman J. D. Robert S. A. (2000). Neighborhood socioeconomic status and perceptions of self-efficacy. Sociol. Perspect. 43, 117–136. 10.2307/1389785

[B17] Bong M. Clark R. E. (1999). Comparison between self-concept and self-efficacy in academic motivation research. Educ. Psychol. 34, 139–153. 10.1207/s15326985ep3403_1

[B18] Breiman L. (2001). Random forests. Mach. Learn. 45, 5–32. 10.1023/A:1010933404324

[B19] Bureau for Policy and Program Coordination (2004). Strengthening Education in the Muslim World: Country Profiles and Analysis. Available at: https://s3.amazonaws.com/berkley-center/040401USAIDStrengtheningEducationMuslimWorld.pdf (accessed November 24, 2022).

[B20] Caraway K. Tucker C. M. Reinke W. M. Hall C. (2003). Self-efficacy, goal orientation, and fear of failure as predictors of school engagement in high school students. Psychol. Sch. 40, 417–427. 10.1002/pits.10092

[B21] Chen T. Guestrin C. (2016). “XGBoost: a scalable tree boosting system,” in KDD'16: Proceedings of the 22nd ACM SIGKDD International Conference on Knowledge Discovery and Data Mining (New York: ACM), 785–794. 10.1145/2939672.2939785

[B22] Church A. T. Teresa J. S. Rosebrook R. Szendre D. (1992). Self-efficacy for careers and occupational consideration in minority high school equivalency students. J. Couns. Psychol. 39:498. 10.1037/0022-0167.39.4.498

[B23] Church M. A. Elliot A. J. Gable S. L. (2001). Perceptions of classroom environment, achievement goals, and achievement outcomes. J. Educ. Psychol. 93, 43–54. 10.1037/0022-0663.93.1.43

[B24] Cohen J. (1988). Statistical Power Analysis for the Behavioral Sciences, 2nd edn. New York: Routledge.

[B25] Colclough C. King K. McGrath S. (2010). The new politics of aid to education: rhetoric and reality. Int. J. Educ. Dev. 5, 451–452. 10.1016/j.ijedudev.2010.03.009

[B26] Coutinho S. (2008). Self-efficacy, metacognition, and performance. N. Am. J. Psychol. 10, 165–172. Available at: https://psycnet.apa.org/record/2008-03556-012

[B27] Davies L. (2016). Security, extremism and education: safeguarding or surveillance? Br. J. Educ. Stud. 64, 1–19. 10.1080/00071005.2015.1107022

[B28] Deng J. Heydarnejad T. Farhangi F. Khafaga A. F. (2022). Delving into the relationship between teacher emotion regulation, self-efficacy, engagement, and anger: a focus on English as a foreign language teachers. Front. Psychol. 13:1019984. 10.3389/fpsyg.2022.1019984 36337515 PMC9627275

[B29] Devi K. Ratnoo S. (2022). Predicting student dropouts using random forest. J. Stat. Manag. Syst. 25, 1579–1590. 10.1080/09720510.2022.2130570

[B30] DeWitz S. J. Woolsey M. L. Walsh W. B. (2009). College student retention: an exploration of the relationship between self-efficacy beliefs and purpose in life among college students. J. Coll. Stud. Dev. 50, 19–34. 10.1353/csd.0.004934409987

[B31] Edgar S. Carr S. E. Connaughton J. Celenza A. (2019). Student motivation to learn: is self-belief the key to transition and first year performance in an undergraduate health professions program? BMC Med. Educ. 19:111. 10.1186/s12909-019-1539-5 30999916 PMC6471892

[B32] Emmons R. A. McCullough M. E. (2003). Counting blessings versus burdens: an experimental investigation of gratitude and subjective wellbeing in daily life. J. Pers. Soc. Psychol. 84, 377–389. 10.1037/0022-3514.84.2.377 12585811

[B33] English M. C. Kitsantas A. (2013). Supporting student self-regulated learning in problem- and project-based learning. IJPBL. 7:6. 10.7771/1541-5015.133929356800

[B34] Erozkan A. (2014). Analysis of social problem solving and social self-efficacy in prospective teachers. Educ. Sci. Theory Pract. 14, 447–455. 10.12738/estp.2014.2.2014

[B35] Ezen-Can A. Boyer K. E. (2014). “Toward adaptive unsupervised dialogue act classification in tutoring by gender and self-efficacy,” in Extended Proceedings of the 7th International Conference on Educational Data Mining (EDM) (London, UK: EDM), 94–100. Available at: https://www.dropbox.com/s/crr6y6fx31f36e0/EDM%202014%20Full%20Proceedings.pdf (accessed June 14, 2024).

[B36] Ferudun S. Onur E. (2018). Humility and forgiveness as predictors of teacher self-efficacy. Educ. Res. Rev. 13, 120–128. 10.5897/ERR2017.344938147025

[B37] Filmer D. Rogers H. Al-Samarrai S. Bendini M. Béteille T. Evans D.. (2018). World Development Report 2018: Learning to Realize Education's Promise. Washington, DC: World Bank Group.

[B38] Froh J. J. Sefick W. J. Emmons R. A. (2008). Counting blessings in early adolescents: an experimental study of gratitude and subjective wellbeing. J. Sch. Psychol. 46, 213–233. 10.1016/j.jsp.2007.03.005 19083358

[B39] Gootman M. E. (2001). The Caring Teacher's Guide to Discipline: Helping Young Students Learn Self-Control, Responsibility, and Respect, 2nd ed. Thousand Oaks, CA: Corwin Press.

[B40] Hair J. F. (2010). Multivariate Data Analysis, 7th ed. Upper Saddle River, NJ: Prentice Hall.

[B41] Hargreaves A. Earl L. Moore S. Manning S. (2001). Learning to Change: Teaching beyond Subjects and Standards. San Francisco, CA: Jossey-Bass.

[B42] Hooshyar D. Pedaste M. Yang Y. (2019). Mining educational data to predict students' performance through procrastination behavior. Entropy. 22:12. 10.3390/e22010012 33285787 PMC7516418

[B43] Huang C. (2013). Gender differences in academic self-efficacy: a meta-analysis. Eur. J. Psychol. Educ. 28, 1–35. 10.1007/s10212-011-0097-y

[B44] James G. Witten D. Hastie T. Tibshirani R. (2013). An Introduction to Statistical Learning. New York: Springer.

[B45] Jin R. Wu R. Xia Y. Zhao M. (2023). What cultural values determine student self-efficacy? An empirical study for 42 countries and economies. Front. Psychol. 14:1177415. 10.3389/fpsyg.2023.1177415 37408968 PMC10319125

[B46] Kagitcibasi C. (2005). Autonomy and relatedness in cultural context: implications for self and family. J. Cross Cult. Psychol. 36, 403–422. 10.1177/002202210527595930152264

[B47] Kapoor I. (2014). Psychoanalysis and development: contributions, examples, limits. Third World Q. 35, 1120–1143. 10.1080/01436597.2014.9261019432524

[B48] Lande N. Ask T. Sætren S. Lugo R. Sütterlin S. (2023). The role of emotion regulation for general self-efficacy in adolescents assessed through both neurophysiological and self-reported measure. PsyArXiv [Preprints]. 10.2147/PRBM.S406702 37650113 PMC10464900

[B49] Lent R. W. Brown S. D. Larkin K. C. (1984). Relation of self-efficacy expectations to academic achievement and persistence. J. Couns. Psychol. 31, 356–362. 10.1037/0022-0167.31.3.356

[B50] Liu J. Wu J. Liu S. Li M. Hu K. Li K. (2021). Predicting mortality of patients with acute kidney injury in the ICU using XGBoost model. PLoS ONE. 16:e0246306. 10.1371/journal.pone.0246306 33539390 PMC7861386

[B51] Ljubic B. Pavlovski M. Gillespie A. Rubin D. Collier G. Obradovic Z. (2022). Systematic review of supervised machine learning models in prediction of medical conditions. medRxiv. 10.1101/2022.04.22.2227418331965266

[B52] McKenzie M. (2012). Education for y'all: global neoliberalism and the case for a politics of scale in sustainability education policy. Policy Futures Educ. 10, 165–177. 10.2304/pfie.2012.10.2.16518307325

[B53] McMahon S. D. Wernsman J. (2009). The relation of classroom environment and school belonging to academic self-efficacy among urban fourth- and fifth-grade students. Elem. Sch. J. 109, 267–281. 10.1086/592307

[B54] McQuiggan S. W. Mott B. W. Lester J. C. (2008). Modeling self-efficacy in intelligent tutoring systems: an inductive approach. User Model. User-Adapt. Interact. 18, 81–123. 10.1007/s11257-007-9040-y

[B55] Merrill K. L. Smith S. W. Cumming M. M. Daunic A. P. (2017). A review of social problem-solving interventions: past findings, current status, and future directions. Rev. Educ. Res. 87, 71–102. 10.3102/003465431665294338293548

[B56] Nasser I. Miller-Idriss C. Alwani A. (2019). Reconceptualizing education transformation in Muslim societies: the human development approach. JEMS. 1, 3–25. 10.2979/jems.1.1.02

[B57] Nasser I. Saroughi M. (2021). Advancing education in Muslim societies: mapping the terrain. JEMS. 2, 90–102. 10.2979/jems.2.2.06

[B58] National Center for Education Statistics (1996). Education Indicators: An International Perspective. Available at: https://nces.ed.gov/pubs/eiip/eiip1s01.asp (accessed March 16, 2024).

[B59] Oliveira Almeida R. Almeida Munis R. Aparecido Camargo D. da Silva T. Sasso Júnior V. A. Simões D. (2022). Prediction of road transport of wood in Uruguay: approach with machine learning. Forests 13:1737. 10.3390/f13101737

[B60] Osterman K. F. (2000). Students' need for belonging in the school community. Rev. Educ. Res. 70, 323–367. 10.3102/0034654307000332338293548

[B61] Pajares F. (1996). Self-efficacy beliefs in academic settings. Rev. Educ. Res. 66, 543–578. 10.3102/0034654306600454338293548

[B62] Pajares F. (2008). “Motivational role of self-efficacy beliefs in self-regulated learning,” in Motivation and Self-Regulated Learning: Theory, Research, and Applications, eds. G. A. D. Liem and D. S. McInerney (Mahwah, NJ: Lawrence Erlbaum), 111–139.

[B63] Peña-Ayala A. (2014). Educational data mining: a survey and a data mining-based analysis of recent works. Expert Syst. Appl. 41, 1432–1462. 10.1016/j.eswa.2013.08.042

[B64] Rakhshanderou S. Safari-Moradabadi A. Ghaffari M. (2021). Structural equation modeling of the spirituality and self-efficacy among college students. J. Relig. Health. 60, 488–499. 10.1007/s10943-020-00984-y 31960356

[B65] Rey D. (2009). The Relationship of Gratitude and Subjective Wellbeing to Self-Efficacy and Control of Learning Beliefs Among College Students [dissertation]. Los Angeles, CA: University of Southern California.

[B66] Rizk N. Farooque A. (2021). “Using K-nearest neighbors to classify undergraduate female self-efficacy in computer science,” in International Academy of Technology, Education and Development (IATED), Valencia, Spain, 71–77. 10.21125/inted.2021.0026

[B67] Roeser R. W. Midgley C. Urdan T. C. (1996). Perceptions of the school psychological environment and early adolescents' psychological and behavioral functioning in school: the mediating role of goals and belonging. J. Educ. Psychol. 88, 408–422. 10.1037/0022-0663.88.3.408

[B68] Ryan R. M. Stiller J. D. Lynch J. H. (1994). Representations of relationships to teachers, parents, and friends as predictors of academic motivation and self-esteem. J. Early Adolesc. 14, 226–249. 10.1177/027243169401400207

[B69] Sahlaoui H. Alaoui E. A. A. Nayyar A. Agoujil S. Jaber M. M. (2021). Predicting and interpreting student performance using ensemble models and Shapley additive explanations. IEEE Access 9, 152688–152703. 10.1109/ACCESS.2021.3124270

[B70] Sahlberg P. Hasak J. Rodriguez V. (2017). Hard Questions on Global Educational Change: Policies, Practices, and the Future of Education. New York: Teachers College Press.

[B71] Saroughi M. Kitsantas A. (2021). Examining relationships among contextual, motivational and wellbeing variables of immigrant language-minority college students. Innov. High. Educ. 46, 1–19. 10.1007/s10755-020-09520-y

[B72] Schunk D. H. (1987). Peer models and children's behavioral change. Rev. Educ. Res. 57, 149–174. 10.3102/0034654305700214938293548

[B73] Schunk D. H. (2005). Self-regulated learning: the educational legacy of Paul R. Pintrich. Educ. Psychol. 40, 85–94. 10.1207/s15326985ep4002_3

[B74] Schunk D. H. Pajares F. (2002). “The development of academic self-efficacy,” in Development of Achievement Motivation, eds. A. Wigfield and J. S. Eccles (San Diego, CA: Academic Press), 15–31.

[B75] Schunk D. H. Zimmerman B. J. (2008). Motivation and Self-Regulated Learning: Theory, Research, and Applications. New York: Routledge.

[B76] Schurz J. (2018). Engaging the Other: Examining How Empathy Facilitates Self-Efficacy [dissertation]. Waco, TX: Baylor University. Available at: https://baylor-ir.tdl.org/handle/2104/10315 (accessed March 11, 2023).

[B77] Schwarzer R. Luszczynska A. (2006). “Self-efficacy, adolescents' risk-taking behaviors, and health,” in Self-Efficacy Beliefs of Adolescents, eds. F. Pajares and T. Urdan (Greenwich, CT: Information Age Publishing), 139–159.

[B78] Simsekler M. C. E. Alhashmi N. H. Azar E. King N. Luqman R. Al Mulla A. (2021a). Exploring drivers of patient satisfaction using a random forest algorithm. BMC Med. Inform. Decis. Mak. 21:157. 10.1186/s12911-021-01519-5 33985481 PMC8120836

[B79] Simsekler M. C. E. Rodrigues C. Qazi A. Ellahham S. Ozonoff A. (2021b). A comparative study of patient and staff safety evaluation using tree-based machine learning algorithms. Reliab. Eng. Syst. Saf. 208:107416. 10.1016/j.ress.2020.107416

[B80] Siu A. M. Shek D. T. (2010). Social problem solving as a predictor of wellbeing in adolescents and young adults. Soc. Indic. Res. 95, 393–406. 10.1007/s11205-009-9527-5

[B81] Slaten C. D. Ferguson J. K. Allen K. Brodrick D. Waters L. (2016). School belonging: a review of the history, current trends, and future directions. Educ. Dev. Psychol. 33, 1–15. 10.1017/edp.2016.6

[B82] Smith S. W. Daunic A. P. (2006). Managing Difficult Behaviors Through Problem Solving Instruction: Strategies for the Elementary Classroom. Boston, MA: Pearson Allyn and Bacon.

[B83] Sobnath D. Kaduk T. Ur Rehman I. Isiaq O. (2020). Feature selection for UK disabled students' engagement post higher education: a machine learning approach for a predictive employment model. IEEE Access. 8, 159530–159541. 10.1109/ACCESS.2020.3018663

[B84] Suha S. A. Sanam T. F. (2022). “A machine learning approach for predicting patient's length of hospital stay with random forest regression,” in 2022 IEEE Region 10 Symposium (Mumbai, India: IEEE), 1–6.

[B85] Tan B. Cutumisu M. (2022). “Employing tree-based algorithms to predict students' self-efficacy in PISA 2018,” in Proceedings of the 15th International Conference on Educational Data Mining (Durham), 634.

[B86] Tawil S. Cougoureux M. (2013). Revisiting Learning: The Treasure within; Assessing the Impact of the 1996 Delors Report. Available at: https://unesdoc.unesco.org/ark:/48223/pf0000220050 (accessed June 8, 2024).

[B87] Ting S.-C. Yeh L.-Y. (2014). Teacher loyalty of elementary schools in Taiwan: the contribution of gratitude and relationship quality. Sch. Leadersh. Manag. 34, 85–101. 10.1080/13632434.2013.813453

[B88] Usher E. L. Pajares F. (2008). Sources of self-efficacy in school: critical review of the literature and future directions. Rev. Educ. Res. 78, 751–796. 10.3102/003465430832145638293548

[B89] Wang Y. Xu L. Qin W. Zhang J. Xia Y. Jing X.. (2019). Gender difference in general self-efficacy among young-old elderly aged 60–74 in rural Shandong China: a cross-sectional survey. Int. J. Environ. Res. Public Health 16:5070. 10.3390/ijerph16245070 31842316 PMC6950069

[B90] Wolters C. A. Hussain M. (2015). Investigating grit and its relations with college students' self-regulated learning and academic achievement. Metacogn. Learn. 10, 293–311. 10.1007/s11409-014-9128-9

[B91] Zimmerman B. J. (1989). A social cognitive view of self-regulated academic learning. J. Educ. Psychol. 81, 329–339. 10.1037/0022-0663.81.3.32910753547

[B92] Zimmerman B. J. (1995). “Self-efficacy and educational development,” in Self-Efficacy in Changing Societies, ed. A. Bandura (Cambridge, UK: Cambridge University Press), 202–231. 10.1017/CBO9780511527692.009

[B93] Zimmerman B. J. (2000). Self-efficacy: an essential motive to learn. Contemp. Educ. Psychol. 25, 82–91. 10.1006/ceps.1999.1016 10620383

